# A review of documents assessing capacity and treatment needs to safeguard adults with incapacity

**DOI:** 10.1192/bjo.2021.255

**Published:** 2021-06-18

**Authors:** Tereza Hoggard, Robin Holliday, Everett Julyan

**Affiliations:** 1 University of Glasgow; 2 NHS Ayrshire and Arran

## Abstract

**Aims:**

To audit the completion of Adults with Incapacity (AWI) documents (Assessment of Capacity, Section 47 Certificate of Incapacity and Treatment Plan) to ensure they met the legal standards required. We hypothesised that the forms were not all completed comprehensively, particularly with regards to the Treatment Plans.

**Method:**

In addition to being legal documents, AWI documents provide an important framework to guide clinicians when giving treatment and balancing patient safety with patient autonomy. Correctly completed documents help provide vulnerable patients with ethical and lawful treatment that allows them to be treated with respect and dignity.

An audit was conducted across two Old Age Psychiatry wards at Ayrshire Central Hospital during October 2020. We assessed all AWI documents available on the wards (n = 20) using criteria based on the standards set by the Mental Welfare Commission for Scotland to ensure legal competence.

**Result:**

95% of the forms were signed and dated, and the nature of the incapacity was given in 100% of the documents. On the other hand, 35% of the forms gave no indication of the presence or absence of a guardian. Only one of those identified as having a guardian was consulted with regards to the treatment plan. Another member of staff was consulted on the Treatment Plan in 45% of cases. 30% of the Treatment Plans were not precisely worded enough to be considered justifiable for treatment. In the Certificate of Incapacity, two out-of-date certificates were found, and staff were notified immediately. 45% of certificates were considered over-generalised with regards to the description under medical treatment.

**Conclusion:**

Overall, the forms were mostly signed and dated, with the nature of incapacity given. The two areas that appeared to be the most problematic were the issue of identifying and discussing plans with a guardian, and the specification of treatment covered by both the Certificate of Incapacity and the Treatment Plan.

Discussion with members of the healthcare team found some confusion over how to complete the forms and many cited a lack of formal training as the main reason for their uncertainty. In addition, accessing clear information online or on the wards on how to complete the forms was challenging. We intend to improve the completion of these documents by implementing teaching and a guidance poster, based on the areas that we identified as being problematic, and completing the audit cycle.

